# Transdiagnostic Internet Intervention for Indonesian University Students With Depression and Anxiety: Evaluation of Feasibility and Acceptability

**DOI:** 10.2196/20036

**Published:** 2021-03-05

**Authors:** Metta Rahmadiana, Eirini Karyotaki, Mieke Schulte, David Daniel Ebert, Jan Passchier, Pim Cuijpers, Thomas Berger, Wouter van Ballegooijen, Supra Wimbarti, Heleen Riper

**Affiliations:** 1 Department of Clinical, Neuro and Developmental Psychology Faculty of Behavioural and Movement Sciences Vrije Universiteit Amsterdam Amsterdam Netherlands; 2 Amsterdam Public Health Research Institute Amsterdam Netherlands; 3 Department of Global Health and Social Medicine Harvard Medical School Harvard University Boston, MA United States; 4 Division of Online Health Training, Innovation Incubator Leuphana University of Lueneburg Lueneburg Germany; 5 Department of Clinical Psychology and Psychotherapy Friedrich-Alexander University Erlangen-Nürnberg Erlangen Germany; 6 Department of Clinical Psychology and EMGO Institute for Health and Care Research Vrije Universiteit Amsterdam Amsterdam Netherlands; 7 Department of Clinical Psychology and Psychotherapy University of Bern Bern Switzerland; 8 Department of Psychiatry Vrije Universiteit Medical Centre/GGZ inGeest Amsterdam Netherlands; 9 Faculty of Psychology Universitas Gadjah Mada Yogyakarta Indonesia

**Keywords:** anxiety, cultural adaptation, depression, guided, internet-based intervention, transdiagnostic, university students

## Abstract

**Background:**

University students with depression and anxiety do not easily receive or seek treatment; therefore, internet-based interventions have been suggested to be a promising way to improve treatment accessibility and availability. However, it has not been examined whether a guided, culturally adapted, transdiagnostic, internet-based intervention is effective for treating symptoms of depression, anxiety, or both among university students in Indonesia.

**Objective:**

This study aims to investigate the feasibility (acceptability and satisfaction, usability, and uptake) of a guided, culturally adapted, transdiagnostic, internet-based intervention among university students with symptoms of depression, anxiety, or both in Indonesia.

**Methods:**

Students from Universitas Gadjah Mada, Yogyakarta, Indonesia, were screened for symptoms of depression, anxiety, or both, and filled online informed consent, demographic questionnaires, and a quality of life measure at pretreatment assessment (T0). Subsequently, the participants started the intervention. Seven weeks after T0, the primary outcomes of this feasibility study were analyzed at posttreatment assessment (T1) using the 8-item Client Satisfaction Questionnaire (CSQ-8) and the System Usability Scale (SUS). Mean and SDs for the CSQ-8 and SUS were calculated to examine feasibility. Within-group secondary outcomes (depression, anxiety, and quality of life) were inspected for outliers and normal distribution. Paired-sample t tests were used to investigate differences between time points of secondary outcomes. A mixed-method approach of quantitative and qualitative analyses was adopted. Both the primary and secondary outcomes were additionally explored with an individual semistructured interview and synthesized descriptively.

**Results:**

A total of 50 participants completed the intervention. We found a moderate to high level of satisfaction and acceptability, a slightly below-average level of desirable usability (≥70), and an adherence rate of 52% which was higher than expected given the novelty of the intervention. Results for the secondary outcomes indicated a decrease in depression and anxiety. For depression, the overall mean difference between the 2 time points for depression was 3.92 (95% CI 2.75-5.1; Hedges *g* 1.15; *P*<.001). For anxiety, the overall mean difference between the 2 time points was 3.34 (95% CI 2.06-4.61; Hedges *g* 1.02; *P*<.001). Further, a moderate effect in improving quality of life was found (*g*=0.50). Overall, participants were positive about the online intervention and ECoaches (online guidance), and they found the intervention to be culturally appropriate.

**Conclusions:**

A culturally adapted, transdiagnostic, internet-based intervention appears to be acceptable and feasible for reducing symptoms of depression, anxiety, or both, and increasing quality of life in university students in Indonesia. Future studies should include a randomized controlled trial to assess the effectiveness of such interventions as they may supplement existing counseling services in universities, reduce the treatment costs, and maximize treatment accessibility in low-resourced settings.

**International Registered Report Identifier (IRRID):**

RR2-10.1016/j.invent.2018.11.002

## Introduction

University students may experience common mental health disorders such as depression and anxiety [1-3]. Epidemiological studies [4] have shown that depression and anxiety are prevalent in college students (31%). This percentage is significantly larger when compared with nonstudents (21.4%) in the same age group [5]. The prevalence of anxiety and depression among university students may be influenced by the complexity of the transition from late adolescence to young adulthood [6]. This common mental health risk has been associated with academic demands [7], social adjustment [8], and financial challenges that students may face [9]. Depression and anxiety can be effectively treated with either pharmacotherapy or psychotherapy [10]. This is in line with the biopsychosocial approach suggested by the World Health Organization (WHO) for managing common mental disorders. The biopsychosocial approach promotes a thorough understanding that mental health issues are an outcome of biological, psychological, and social factors [11]. Effective treatment is imperative as it may prevent a series of negative cascades, such as poor academic adjustment, study dropout, and chronicity [12,13]. Many universities have counseling services that play an important role in improving mental health care by providing effective, accessible, and confidential ways to detect, prevent, and treat common mental disorders in students. The effectiveness of university counseling services for students with high levels of distress has been reported [14]. However, most university students do not receive or seek treatment at counseling services for their psychological problems [6] due to stigma, lack of time, lack of motivation, or they have a preference for a self-management approach [15]. In addition, the low university mental health service availability and high underutilization are very common in non-Western countries [16,17]. In Indonesia, university counseling services are unevenly distributed across universities, as they are mostly dependent on the presence and service of faculties of psychology in the universities.

Therefore, novel therapeutic delivery formats for university students with depression and anxiety may be a feasible way to improve treatment accessibility. Digital technologies offer an excellent opportunity for improving treatment availability and accessibility, especially in low- and middle-income countries, given the widespread acceptance of internet and mobile phone use among university students. Studies on interventions delivered via the internet have shown promising outcomes for students in Western countries due to their anonymity, accessibility, and adaptability to student needs [18,19]. Another known characteristic of internet-based interventions is that they can also be delivered with some form of human guidance (eg, by an ECoach) or can be purely self-guided [20]. Guided internet-based interventions were reported to be more effective compared to self-guided interventions, possibly because participants receive feedback and motivation, and this encourages them to proceed with the sessions and understand the content of the intervention better [21].

For Indonesian university students, internet-based interventions may have a high level of feasibility for a number of reasons. First, internet use is widespread among Indonesian university students (74.23% use the internet), compared to the general Indonesian population, with 94% of them using the internet on their mobile phones [22]. Second, guided internet-based interventions may be more attractive than face-to-face treatment in and outside the university, as the latter involves a high cost that might not be affordable for Indonesian students. Thus, internet-based interventions may be more favorable because they include minimal asynchronous support at a much lower cost than face-to-face interventions. Besides, such interventions can be accessed anywhere [20,23], at any time, and are thus more flexible for students who have very demanding daily schedules at the university.

Third, online interventions can reduce the fear of being exposed to others because students can access the online interventions anonymously [23]. By contrast, students may not utilize internet interventions for a number of reasons including time constraints due to their study duties, lack of nonverbal communication with the ECoach, and the high demand of self-discipline associated with self-help interventions [24]. Fourth, in Indonesia, internet-based interventions for depression and anxiety are currently not available for either university students or for the general population. The first randomized control trial on internet-based behavioral activation for adult depression in Indonesia [25,26] showed promising results with lower symptoms at posttest assessment in favor of the intervention with minimum support by a lay counselor who did not have professional education and qualifications in mental health care. However, the intervention was tested in the general population, and thus, it remains unclear whether these interventions are also effective in university students with elevated symptoms of depression and anxiety. Additionally, given that depression and anxiety are highly comorbid conditions [27,28], a transdiagnostic approach could be beneficial as it targets both symptoms of depression and anxiety simultaneously. Findings on transdiagnostic interventions for university students in Western countries with common mental disorders have been encouraging. More specifically, such interventions have shown to reduce symptoms of common mental disorders with moderate to large effect sizes in the range of 0.42-0.80 for outcomes such as depression and anxiety [23,29-31].

To the best of our knowledge, it has not yet been examined whether guided, culturally adapted, transdiagnostic, internet-based interventions are feasible in treating symptoms of depression or anxiety or both among university students in non-Western countries, such as Indonesia. Therefore, in this pilot study, we aimed to investigate the feasibility (defined as acceptability, satisfaction, usability, and uptake) of a guided, culturally adapted, transdiagnostic internet-based intervention among university students with symptoms of depression, anxiety, or both in Indonesia. This paper reports the results of stage 3 from a theory-based cultural adaption framework, namely, preliminary adaptation test [32]. The first 2 stages of the framework, namely, information gathering and preliminary adaptation design, are reported in the protocol paper of this study [33].

## Methods

### Participants

Participants were recruited via our study website [34], which was disseminated on posters, social media platforms, and business cards containing brief information about the study. As we described in our protocol [33], there is no golden standard for calculating the sample size of a pilot study [35]. For our study, we performed a post hoc power calculation to describe statistically the power of our chosen sample (n=50 participants). Further details can be found in our study protocol [33]. Participants were eligible if they (1) were students at Universitas Gadjah Mada Yogyakarta with access to broadband internet, (2) were 18 years of age or older, (3) could speak and read Bahasa Indonesia fluently, and (4) experienced mild to moderate depression, anxiety, or both (9-item Patient Health Questionnaire [PHQ-9] score >4, 7-item Generalized Anxiety Disorder [GAD-7] score >4, or both). Exclusion criteria were (1) moderately severe depression, anxiety, or both (PHQ-9 score >14, GAD-7 score >14, or both) and (2) currently receiving psychological treatment for depression, anxiety, or both.

### Procedures

Students who were interested in participating in the pilot study filled in their demographic details on the digital registration form on our study website. Subsequently, the students received an email with a link, which directed them to the pretreatment assessment (T0) consisting of the study’s screening questionnaires: the Indonesian version of the PHQ-9 [36,37] and the GAD-7 scale [36,37]. After completing the PHQ-9 and GAD-7 at the screening, participants were asked to provide an informed consent for using their responses to these questionnaires in our analyses (retrospectively). For pragmatic reasons, these eligibility screening scores were also used as a baseline assessment for included participants. Subsequently, an information sheet was provided to them through an emailed link containing an explanation of the study and an online informed consent form that needed to be signed before being able to participate. Those who consented to participate received additional questions about their demographics and quality of life using the Indonesian version of the Euro Quality of Life 5 Dimension-5 Level Scale (EQ5D5L) [38]. The EQ5D5L was used because performance and changes in mental health are captured through one of its dimensions (depression/anxiety) and it is a widely used generic quality of life scale that is also usable for health economic calculations. After completing these additional questionnaires, participants were assigned online to an ECoach (a trained Clinical Psychology Master’s student from the Faculty of Psychology at Universitas Gadjah Mada or a licensed Psychologist) and were given secure login details. Subsequently, the I-AiMentalWELLness (*Saya menuju mental sehat*) online intervention was activated for them through Mind District [39], an eHealth platform providing digital therapy.

The posttreatment assessment (T1) was given 7 weeks after the pretreatment assessment (T0) and consisted of the primary outcomes of this feasibility study, namely, participants’ reported acceptability and satisfaction, usability, and uptake.

Further details about the procedures of this pilot study can be found in our protocol paper [33]. Ethical approval was obtained from the Medical and Health Research Committee of the Medical Faculty in Universitas Gadjah Mada/DR Sardjito General Hospital (reference number: KE/FK/0098/EC/2018).

### Measures

#### Primary Outcomes

The participants’ reported acceptability and satisfaction, as well as the usability and uptake of the I-AiMentalWELLness (*Saya menuju mental sehat*) intervention were the primary outcomes of this feasibility study. Acceptability and satisfaction were measured using the Indonesian version of the 8-item Client Satisfaction Questionnaire (CSQ-8) [40]. Moreover, we used the Indonesian version of the System Usability Scale (SUS) [41] as a usability outcome. While the psychometric properties for the Indonesian version of the CSQ-8 and the SUS are currently not available, both had good psychometric properties in studies conducted in Western countries (CSQ-8 and SUS Cronbach =.93 and .90, respectively) [42-44]. The internal consistency for CSQ-8 and SUS in this study was good with Cronbach α=.87 and .83, respectively. Moreover, the CSQ-8 has been adapted to internet-based interventions (CSQ-I) and has demonstrated overall good psychometric properties in 2 studies [45].

In the study protocol, we reported that intervention uptake would be measured by participants’ adherence to the intervention. Adherence was defined as the number of log-ons, time spent on site, and number of sessions attempted. However, due to the technical limitations of the platform used, we could not measure adherence using the numbers of log-ons and time spent online as we originally intended to do [33]. Thus, in this paper, we have described adherence as the number of participants who completed the sessions in the online intervention divided by the number of participants who started the intervention [46]. We also calculated the initial uptake rates by dividing the number of participants who responded to the invitation link from the number of participants who initially expressed interest in participating in the study. We did so to give an overall impression about how many students remained interested in doing the intervention when they had the option to do so.

#### Secondary Outcomes

Secondary outcome measures included depression, anxiety, and quality of life, which were measured using PHQ-9, GAD-7, and EQ5D5L, respectively. The psychometric properties for the Indonesian version of PHQ-9 and GAD-7 are currently not available. Nonetheless, both PHQ-9 and GAD-7 have demonstrated good psychometric properties in studies conducted in Western countries. The PHQ-9 had high internal consistency (α=.89) and a reliability value of 0.84 [47]. Meanwhile, GAD-7 indicated good internal consistency (α=.92) and test–retest reliability of 0.83 [48]. The internal consistency for PHQ-9 was on the edge of being satisfactory (Cronbach α=.70) and that for GAD-7 was good (Cronbach α=.83). The test–retest reliability of the Indonesian version of EQ5D5L was assessed with sequential measurements using the weighted kappa. Results indicated fair agreements of 0.35, 0.30, 0.37, and 0.39 for the dimensions of mobility, self-care, usual activities, and anxiety/depression, respectively [49]. Meanwhile, the intraclass correlation coefficient for Visual Analog Scale was reported as 0.32, indicating a moderate value [49].

#### Semistructured Interviews

Both the primary and secondary outcomes of this feasibility study were explored additionally with an individual semistructured interview to obtain a broader perspective and realistic understanding of acceptability, satisfaction, and usability concerning the culturally adapted online intervention for our student population. The semistructured interviews were conducted after participants completed the interventions, by videoconferences between the first author (MR) in The Netherlands and 10 participants in Indonesia. We took account of heterogeneous factors by ensuring equal representation of age, gender, educational, and ethnic background to gain a better understanding of the primary outcome. The demographic characteristics of the participants included in the semistructured interview are reported in Table 1.

**Table 1:** Demographic characteristic of participants included in the semistructured interview (N=10).

Characteristics		Value	
Age (years), mean (SD); range		24.5 (6.73); 19-41	
**Gender, n (%)**			
	Female	6 (60)	
	Male	4 (40)	
**Ethnicity, n (%)**			
	Java	8 (80)	
	Other	2 (20)	
**Level of education, n (%)**			
	Bachelor	6 (60)	
	Doctorate	1 (10)	
	Master	3 (30)	
**Study program, n (%)**			
	Psychology	3 (30)	
	Medical	2 (20)	
	Other	5 (50)	

The interview questions were modified from the study of Devi et al [50] to fit the purpose of this study (Multimedia Appendix 1). The participants’ responses were audio-recorded, and additional note taking was also performed during data collection by the first author and 1 team member (local supervisor) in Indonesia. The interview time range was approximately 30 minutes and was conducted in Bahasa Indonesia.

The secondary outcomes were further explored to understand (1) each participant’s evaluation of his/her ECoach, and (2) the cultural appropriateness of the adapted internet-based intervention. These interview questions were modified [51] to fit the purpose of the study. The interview questions can be found in Multimedia Appendix 1.

### The I-AiMentalWELLness Intervention

This intervention targets common cognitive and behavioral processes of both anxiety and depression across all sessions; therefore, it is considered a transdiagnostic intervention. Moreover, this intervention was based on cognitive behavioral therapy principles [51,52]. It was originally developed for the general population in Germany and Switzerland [53] and then adapted to meet the needs of domestic and international university students in The Netherlands [54,55]. For the purpose of this study, the current version was culturally adapted from English to Bahasa Indonesia for the student population based on the heuristic theoretical framework [32] which entails (1) information gathering, (2) preliminary adaptation design, (3) preliminary testing, (4) adaptation refinement, and (5) cultural adaptation trial. As reported in the study protocol [33], the first and second phase involved end users (students) as part of the developmental process of the current intervention. Further, the cultural adaptation process concerned all elements of the intervention including language, images, testimonials, and other examples that might not be applicable to the Indonesian culture. Core components of the intervention (eg, behavioral activation, problem solving, cognitive restructuring) remained unchanged. More specifically, after the focus group discussions, we omitted all parts related to unmarried cohabitation, sexual activities, and terms related to alcoholic beverages due to the majority of university students being Muslim. These topics are considered inappropriate in the Islamic religion. We have also changed all parts related to medication such as antidepressants and sleep medication, winter-related sports, and membership in sports and music school. These culturally adapted changes did not influence the main therapeutic components of the internet-based cognitive behavioral therapy intervention (eg, cognitive restructuring) but they solely reflected the local context. An image of one of the sessions in the I-AiMentalWELLness (*Saya menuju mental sehat*) intervention is provided in Multimedia Appendix 2.

The online intervention consisted of 7 sessions and an additional booster session, which took place 4 weeks after the seventh session of the intervention was completed. The purpose of the booster session was to refresh the memory of the participants about what they learned in the previous 7 sessions of the intervention. It aimed at reinforcing the progress that has been made throughout the interventions and to prepare in case the participants encounter a new episode of, for example, depressive mood. The transdiagnostic approach for anxiety or depression is found particularly in sessions 5 and 6. For further detailed description of the intervention’s content, the reader is referred to Table 2.

**Table 2:** The content of the intervention.

Module	Content description
Session 1: Identifying my needs	Goal setting and behavioral activation
Session 2: Taking action	Problem solving
Session 3: Worth knowing	Psychoeducation on depression and anxiety
Session 4: Thoughts pattern	Cognitive restructuring
Session 5: Dealing with challenges	Solve problems concerning depressive symptoms or exposure to anxiety-provoking situations
Session 6: In daily practice	Solve problems concerning depressive symptoms or exposure to anxiety-provoking situations
Session 7: Future plan	Planning for the future
Session 8: Strong going forward	Booster session
OM^a^ 1: Sleep	Information related to sleep or how to sleep better
OM 2: Perfectionism	Identify high standards and understanding the vicious cycle of perfectionism
OM 3: Gratitude and appreciating good things	Express gratitude and appreciate goodness in life
OM 4: Self-worth	Understanding low self-worth and how to increase it
OM 5: Relaxation	Progressive muscle relaxation
OM 6: Acceptance	Learning to accept unfulfilled needs
OM 7: What is brooding and when is it excessive?	Information on rumination and learning how to overcome it

^a^OM: optional modules.

Each session consisted of text, exercises, testimonials, and audio recordings with a duration of approximately 60 minutes to complete. A monetary incentive of Indonesian Rupiah (IDR) 125.000 (equivalent to €7/US $8.5) was given to participants who completed sessions 1-4 and another IDR 125.000 (equivalent to €7/US $8.5) was given to participants who completed sessions 5-8. This monetary incentive was meant to compensate the prepaid cards that participants used to access internet via their mobile phones. Participants were only allowed to complete a maximum of 2 sessions per week to have enough time to integrate the skills acquired from the intervention into their daily life. After the completion of each session, participants received individualized asynchronous feedback from their ECoach through the messaging system of the platform. According to our protocol, each ECoach was advised to spend a maximum of 30 minutes to give feedback per participant at the end of each session. The feedback was brief and of motivational nature to encourage participants to continue with the intervention. Participants were able to move to the next session only after reading the feedback of their ECoach. Furthermore, participants were allowed to contact their ECoach whenever they wanted via the messaging system in the platform. In such case, the ECoaches were advised to respond within 48 hours. When the feedback had been read by the participant, subsequently, the ECoach unlocked the next intervention session. The feedback was of a motivational nature, meaning it consisted of positive reinforcement and encouragement, which reflected on participants’ responses to the online exercises and homework assignments.

### Statistical and Descriptive Analysis of Qualitative Data

Quantitative analyses were conducted using the IBM SPSS version 25. Because the primary outcomes regarding acceptability, satisfaction, and usability were only assessed after completion of the interventions, these were analyzed only at posttreatment assessment. We calculated means and SD for the CSQ-8 and SUS to display feasibility. Descriptive statistics were used to summarize (1) participants’ demographic information (age, gender, employment, ethnicity, marital status, study program, and level of education) and (2) participants’ uptake.

For the quantitative analyses of within-group secondary outcomes, data were inspected for outliers and normal distribution. In order to investigate differences between pre- and post-assessment of secondary outcomes, paired-sample *t* tests were used. To investigate baseline differences between completers and dropouts, an independent sample *t* test or a Mann–Whitney *U* test was used. Effect sizes were measured using Hedges *g*, and interpreted as small=0.2, medium=0.5, and high effect=0.8 [56].

In the protocol, we suggested performing thematic analysis according to the guidelines [57] to analyze the transcripts of the interviews with the participants. However, the data were not suitable for this approach because the participants did not elaborate in depth on the questions asked. Thus, we did not analyze the results of the interview but instead we descriptively synthesized the qualitative data for both primary (acceptability, satisfaction, and usability regarding the internet-based intervention) and secondary outcomes (ECoach evaluation and cultural appropriateness of the adapted internet-based intervention).

## Results

### Participants

Of the 240 participants who expressed an interest in participating in the study, 118 did not further respond to the invitation link to participate in the screening (initial uptake=50.8%, 122/240). Of those (n=122) screened for eligibility using the PHQ-9 and GAD-7, 96 were eligible to participate in this study and 26 were excluded after obtaining scores indicating moderately severe to severe or no depression or anxiety. Participants with severe depression or anxiety (n=11) were referred to student counseling services, while those who scored below the mild threshold for depression and anxiety (n=15) were excluded and thanked for their participation and interest in our study. The remaining 96 participants were subsequently invited to provide online informed consent, and they completed the additional quality of life measurement (EQ5D5L) and started the first session (intervention uptake=40.0%, 96/240). Fifty participants completed all 7 sessions and the posttreatment assessment. Almost all participants retained at postassessment also followed the booster session (n=46) 4 weeks after the seventh session.

**Figure 1:**
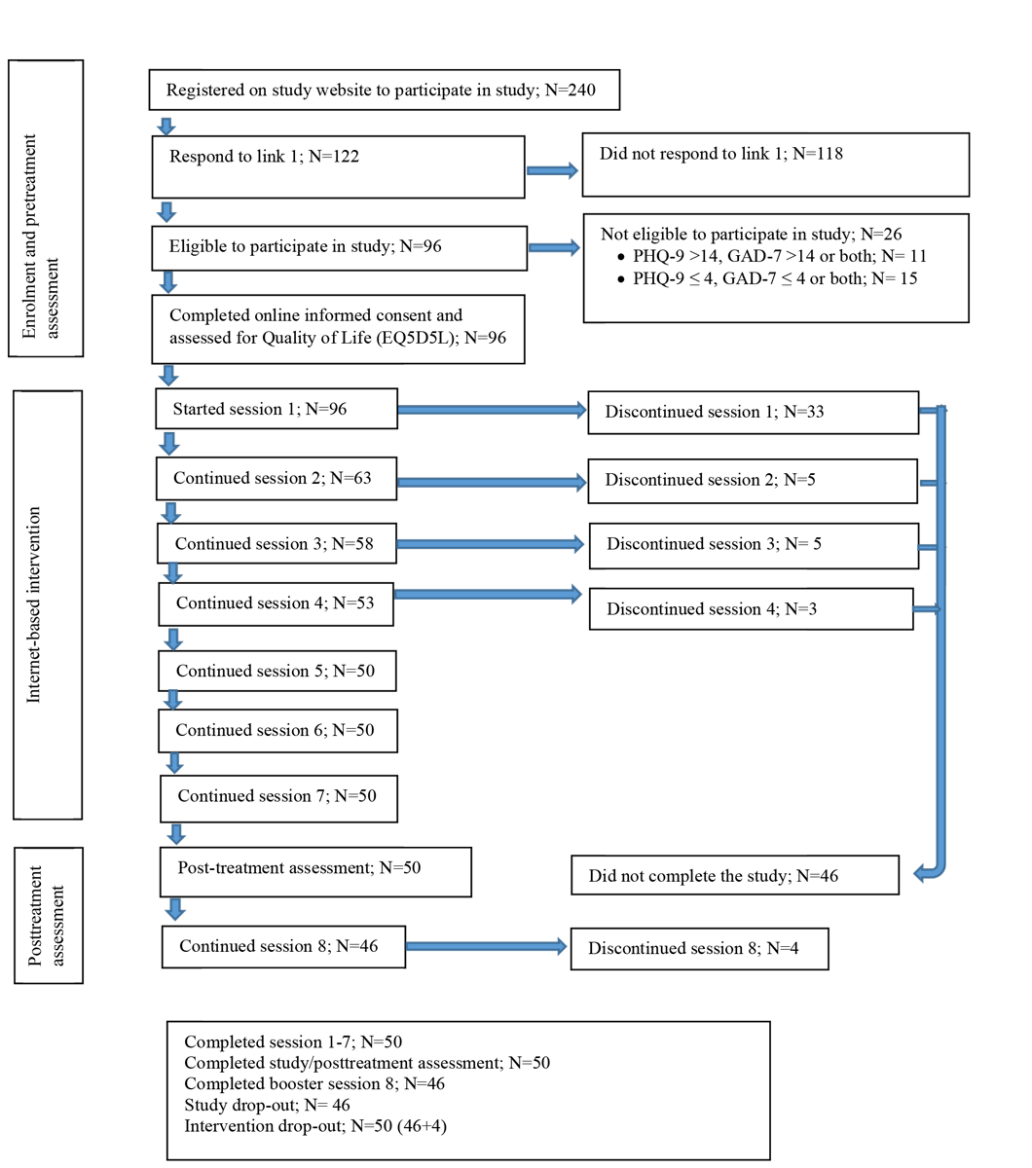
The flowchart of study participants.

A total of 46 participants discontinued the intervention during sessions 1-4. On the Minddistrict platform, the ECoaches were able to view their participants’ platform, enabling them to notice inactivity such as unread feedback or no further progress on a session. If such inactivity was observed, the ECoach made further attempts to contact the participants through email and text messages (eg, SMS text messages or WhatsApp). If the ECoach received no response from inactive participants, they were considered to have discontinued the intervention. In the end, the participants who discontinued (n=46) were not contacted again for the posttreatment assessment because all previous attempts at communication had failed. Last, 50 participants dropped out from the intervention (sessions 1-7 and booster session 8) and 46 participants dropped out from the study. The flow chart of study participants is presented in Figure 1.

The demographic characteristics of the participants and baseline differences between completers and dropouts are presented in Table 3. There were no significant differences in baseline assessment between completers and dropouts (see also Table 3).

**Table 3:** Participants’ characteristics and baseline differences between completers and dropouts.

Characteristics			Completers (N=50)		Dropout (N=46)		*t* (*df*)/ꭓ^2^ (*df*)		*P* value		95% CI
Age (years), mean (SD)			22 (3.74)		22 (5.0)		0.11 (94)^a^		.91		
**Gender,** **n (%)**							0.003 (1)^b^		.96		
	Female	40 (80)		37 (80)							
	Male	10 (20)		9 (20)							
**Ethnicity, n (%)**							0.85 (1)^b^		.36		
	Java	44 (88)		43 (93)							
	Other	6 (12)		3 (7)							
**Marital status, n (%)**							1.62 (2)^b^		.44		
	Divorced	1 (2)		0 (0)							
	Married	3 (6)		5 (11)							
	Single	46 (92)		41 (89)							
**Occupational status,** **n (%)**							0.53 (1)^b^		.47		
	Employed	7 (14)		9 (20)							
	Unemployed	43 (86)		37 (80)							
**Level of education,** **n (%)**							0.50 (2)^b^		.78		
	Bachelor	40 (80)		35 (76)							
	Doctorate	1 (2)		2 (4)							
	Master	9 (18)		9 (20)							
**Study** **program,** **n (%)**							2.68 (1)^b^		.10		
	Psychology	21 (42)		12 (26)							
	Other	29 (58)		34 (74)							
PHQ-9^c^, mean (SD)			9.46 (3.59)		9.33 (3.41)		0.00 (94)^a^		.99		–0.06 to 0.06
GAD-7^d^, mean (SD)			7.54 (3.8)		7.37 (3.46)		–0.22 (94)^a^		.82		–1.65 to 1.30
EQ5D5L^e^, mean (SD)			0.83 (0.10)		0.86 (0.12)		916.5^f^		.08		

^a^Independent sample *t* test.

^b^Chi-square test.

^c^PHQ-9: 9-item Patient Health Questionnaire.

^d^GAD-7: 7-item Generalized Anxiety Disorder.

^e^EQ5D5L: Euro Quality of Life 5 Dimension-5 Level Scale.

^f^Mann–Whitney *U* test.

### Primary Outcomes

The participants who completed the intervention (N=50) had a mean score of 25.8 (SD 3.40) on CSQ-8, indicating moderate to high level of satisfaction and acceptability regarding the online intervention. They had a mean score of 65.1 (SD 13.37) on SUS, indicating a slightly below average of the desirable usability of 70 or more [58]. Of the 96 participants who started, only 50 completed the intervention sessions, resulting in a 52% adherence rate.

### Secondary Outcomes

There was a significant decrease in the PHQ-9 and GAD-7 scores. For depression, the overall mean differences in PHQ-9 between the 2 time points was 3.92 (95% CI 2.75-5.1; Hedges *g* 1.15; *P*<.001). For anxiety, the overall mean differences between the 2 time points in GAD-7 was 3.34 (95% CI 2.06-4.61; Hedges *g* 1.02; *P*<.001), as reported in Table 4. Regarding the EQ5D5L scores, there appeared to be an outlier. However, excluding this outlier did not affect the results, therefore the participant was not excluded from the analysis. At posttreatment assessment, there was a significant increase (*P*=.008) in the EQ5D5L scores, indicating improved quality of life (Table 4).

**Table 4:** Pretreatment and posttreatment assessment for PHQ-9, GAD-7, and EQ5D5L.

Variable	T0, mean (SD)	T1, mean (SD)	*t* (*df)*/Z	*P* value	Effect size (*g*)	95% CI
PHQ-9^a^	9.46 (3.59)	5.54 (3.21)	6.75 (49)^b^	<.001	1.15	2.75-5.1
GAD-7^c^	7.54 (3.8)	4.2 (2.65)	–4.22^d^	<.001	1.02	2.06-4.61
EQ5D5L^e^	0.83 (0.10)	0.88 (0.10)	–2.66^d^	.008	0.50	

^a^PHQ-9: 9-item Patient Health Questionnaire.

^b^Paired sample test.

^c^GAD-7: 7-item Generalized Anxiety Disorder.

^d^Wilcoxon signed-rank test.

^e^EQ5D5L: Euro Quality of Life 5 Dimension-5 Level Scale.

### Descriptive Analysis of the Qualitative Data

Overall, participants (n=9) reported to be satisfied when using the online intervention. It was reported to be informative, challenging, and triggered them to further recognize the symptoms of depression and anxiety as a student. The optional module was rated as being the most enjoyable part of the intervention as it provided many personal insights. The recorded relaxation was also in favor as it delivered a calm feeling. Participants further acknowledged the online intervention to be more practical than face-to-face interventions as it overcame geographical and stigma barriers. Only one participant reported being confused for not receiving an immediate response to questions that might arise when working through the sessions.

Good user experience with the intervention was reported, indicating clarity in the instructions and workflow in a session. However, difficulties were also reported as 1 participant felt unfamiliar with reading online content, whereas 2 others had problems with the stability of the internet connection when working on the intervention in a remote area.

Regarding the semistructured interviews, 6 out of 10 participants responded positively on being supported through the internet by an ECoach as they received sufficient support, encouragement, reminders, and had their work progress monitored. Nonetheless, 4 out of 10 participants were less positive about having an ECoach because of the unavailability of synchronous feedback and having difficulty with writing what is on their mind. Moreover, they reported that the likelihood of being misinterpreted was high because of absent nonverbal communication signals. Finally, feedback was written in a generic manner and did not always cover specific tasks, as these participants would have expected the feedback to be more elaborative.

As much as 7 out of 10 participants indicated that the overall content of the intervention was culturally appropriate and relevant to university students in Indonesia. Nonetheless, 3 out of 10 participants indicated that some case examples of the intervention were less relevant to the Indonesian context. For instance, it is uncommon for Indonesian university students to have a part-time job during bachelor studies or to be enrolled in a sport and music academy.

Some talk about working. Not all students work, especially bachelor students.

There were some story examples that seem less relevant to the Indonesian culture, such as joining a music or a sport club.

## Discussion

### Principal Findings

This study has demonstrated promising outcomes for the feasibility of an internet-based intervention for university students in Indonesia, as indicated by participants’ reported acceptability, satisfaction, usability, and uptake regarding the intervention. In addition, a decrease in symptoms of depression and anxiety, and improved quality of life were reported.

Participants reported to have a moderate to a high level of satisfaction and acceptability regarding the online intervention. Results from the interviews also indicated that participants were satisfied with the online intervention as a new approach that acknowledged personal development, and overcame stigma and geographical barriers. The usability/functionality of the internet-based intervention as it applies to the end users was also assessed. One component of usability explained [41] is learnability, which assesses first-time experiences with a new system for end users. A transdiagnostic approach is characterized by a focus on common characteristics that may underpin a number of different psychological disorders such as in case of depression and anxiety disorders. Advantages of such a treatment approach include treating simultaneously these underlying common factors in case of comorbid symptoms [59]. In light of our positive findings regarding the reduction in anxiety and depressive symptoms, the transdiagnostic approach appears promising in the given context. Further research is needed to investigate the effectiveness of such approach in a randomized control trial. However, a transdiagnostic approach delivered online to treat symptoms of depression and anxiety under a single protocol is relatively new. The findings for the mean usability rate for the internet intervention are slightly below average (65.1) for the desired usability ≥70 [58] which is not surprising. Thus, further development and continued improvement are needed to provide individually tailored transdiagnostic intervention, such as by minimizing the use of formal language in the intervention.

With respect to the secondary outcomes of this study, we found a significant decrease (*P*<.001) in symptoms of depression and anxiety at the posttreatment assessment, and a significant increase (*P*=.008) in quality of life. Online interventions may offer a promising treatment solution, given the high prevalence of common mental disorders among university students and the under-resourced counseling services [1,2,10]. Many studies have indicated the effectiveness of an online intervention to treat depression and anxiety among university students [10,19]. Understanding quality of life among university students is also important, as it contributes to their physical, psychological, social, and environmental well-being [60]. As the quality of life encompasses various dimensions closely related to the life of a university student, it is important that symptoms of depression and anxiety are treated properly.

It must be noted, of course, that our study did not have a control group. Therefore, the improvements might be attributed to other factors than the intervention, such as spontaneous recovery or regression toward mean.

All participants of this feasibility study were guided by an ECoach who monitored their progress throughout the intervention. The likelihood of completion rate may be associated with therapeutic guidance [42,58,60]. However, we have not tested this hypothesis, and this remains to be confirmed by future studies in low-resource settings.

The secondary outcome of this intervention was further supported by the interview results, which indicated participants’ overall positive response to being treated through the internet by an ECoach, the extent of support received, the average time with regard to receiving feedback, and how comprehensively the feedback was delivered.

A randomized controlled trial will follow this promising feasibility study to further investigate the effectiveness of the existing internet-based intervention to treat depression and anxiety for students within university setting in Indonesia. This is a necessary step before this intervention is made publicly available in Indonesia.

### Strengths and Limitations

To the best of our knowledge, this study was one of the first to investigate the feasibility of a guided culturally adapted internet-based intervention to treat symptoms of depression, anxiety, or both among university students in Indonesia. Originally, in the study protocol, we defined appropriate uptake as an adherence rate of 35% due to the novelty of the intervention in Indonesia. However, participants’ adherence rates reached 52% (50/96), which is higher than expected and underlines the feasibility of online interventions for Indonesian university students. It should be noted that we provided monetary incentives to participants who completed the sessions. Thus, it is possible that these incentives have enhanced participation rates. Nevertheless, the amount of incentives was very small and thus it is unlikely that participants completed the study only because of this reason. This feasibility study also has several limitations that must be taken into account. First, the majority of the participants were female, bachelor students, Javanese, and were studying psychology. Future research could benefit from investigating more diverse groups from other faculties and universities to improve the generalizability of these preliminary findings. Second, 46 participants could not be invited to undergo the posttreatment assessment, because they could not be contacted by the ECoach by any means. The research team were unable to locate these participants and thus it limits the understanding of reasons for dropout. Third, data regarding participants’ uptake (log-on time, time spent on site, and number of sessions attempted) of the online intervention could not be provided in this study as participants had previously been instructed to record the time that they spent online. They did so to avoid confusion because Minddistrict (a Dutch company) displayed the Central European time zone. However, this self-reported approach was considered subjective. Other attempts were made by contacting the platform provider to obtain the participants’ recorded time of log-ons but such information was not monitored by the platform. Fourth, the findings regarding the secondary outcome within 2 different time points need to be interpreted with caution as this study was a small pre–post assessment and not a randomized controlled trial. Fifth, another limitation of our study is that our qualitative data were limited in depth, and thus not well suited to thematic analysis. This might have been the result of the way we phrased these questions. In particular, some questions may have been formulated in a too closed manner, thereby preventing the respondents from giving more elaborate answers and convey possible nuances that qualitative research requires. However, the aim of this part of our study was to enrich our quantitative findings. Thus, future qualitative research in this field should aim at more open-ended formulated questions if the aim is to gain in-depth insights into the topic.

### Conclusion

Internet-based interventions appear to be acceptable and feasible for reducing symptoms of anxiety and depression and improving quality of life among university students in Indonesia. Future studies should further investigate the (cost-) effectiveness of such interventions as they may be used to supplement existing counseling services in universities, reduce the treatment costs, and maximize treatment accessibility in low-resourced settings.

## Appendix

Multimedia Appendix 1 Semi-structured interview questions.

Multimedia Appendix 2 Screenshot example of intervention session 3 in Bahasa Indonesia.

## Abbreviations

CSQ 8: Client Satisfaction Questionnaire-8
EQ5D5L: Euro Quality of Life 5 Dimension-5 Level Scale
GAD 7: Generalized Anxiety Disorder-7
IDR: Indonesian Rupiah
PHQ 9: Patient Health Questionnaire-9
SUS: System Usability Scale
