# Supplementary material for: Transdiagnostic Internet Intervention for Indonesian University Students With Depression and Anxiety: Evaluation of Feasibility and Acceptability
Source: JMIR Ment Health. 2021 Mar 5;8(3):e20036. doi: 10.2196/20036 (PMC7980121; doi:10.2196/20036)
Supplement: Multimedia Appendix 1 [file mental_v8i3e20036_app1.pdf]

Multimedia Appendix 1: Semi-structured interview questions

A. Acceptability, satisfaction and usability regarding the cultural adapted online intervention

1. How do you rate the intervention delivered through Internet?
2. Were there any enjoyable parts of the program?
3. Was the Internet-based intervention easy to use? Why yes or why not?

B. Participants' evaluation of their experience with their ECoach

1. How did you experience being treated through the Internet instead of face-to-face interaction?
2. To what extent was the ECoach supportive?
3. How long did it take the ECoach to give feedback?
4. How communicative was the ECoach?
5. How comprehensive was the feedback from the ECoach?

C. Cultural appropriateness of the intervention

1. Were the goals of this Internet session clearly defined?
2. What did you learn from this session?
3. Was the content from this online session clear and easy to understand?
4. Was this online session easy to navigate?
5. Was the length of this session appropriate to the topic?
6. How were the illustrated pictures in this online session?
7. Do you understand the language, idiom, and words used in this module?
8. Do you think the case examples given in this module are appropriate for Indonesian university students?
