# Supplementary figures and images for: Transdiagnostic Internet Intervention for Indonesian University Students With Depression and Anxiety: Evaluation of Feasibility and Acceptability
Source: JMIR Ment Health. 2021 Mar 5;8(3):e20036. doi: 10.2196/20036 (PMC7980121; doi:10.2196/20036)

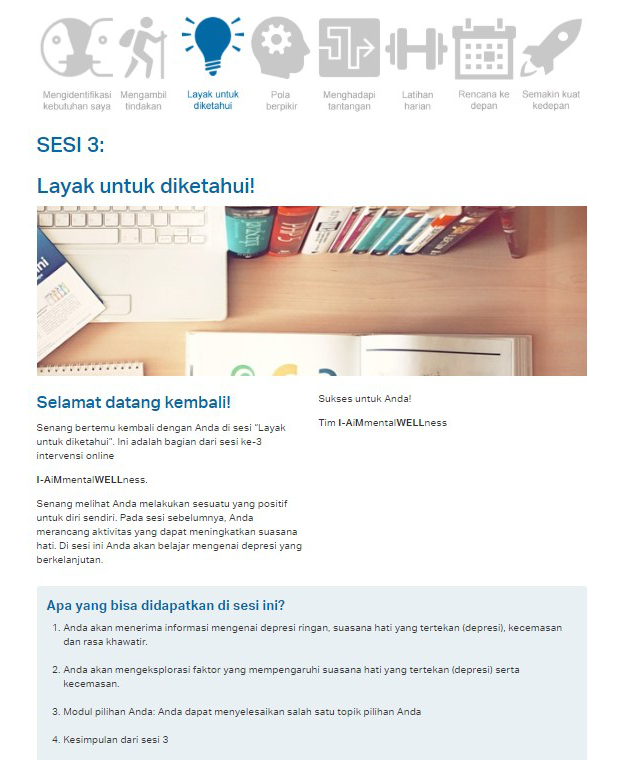

Supplement: Multimedia Appendix 2 [file mental_v8i3e20036_app2.png]
